# Impact of Pb on *Chlamydomonas reinhardtii* at Physiological and Transcriptional Levels

**DOI:** 10.3389/fmicb.2020.01443

**Published:** 2020-06-26

**Authors:** Canqi Zheng, Muhammad Aslam, Xiaojuan Liu, Hong Du, Xihui Xie, Haojie Jia, Nan Huang, Kaiming Tang, Yingquan Yang, Ping Li

**Affiliations:** ^1^Institute of Marine Sciences, Guangdong Provincial Key Laboratory of Marine Biotechnology and STU-UNIVPM Joint Algal Research Center, College of Sciences, Shantou University, Shantou, China; ^2^Southern Marine Science and Engineering Guangdong Laboratory, Guangzhou, China

**Keywords:** physiological responses, Pb toxicity, transcription regulation, hormone, molecular marker

## Abstract

Trace elements stress is one of the most damaging abiotic stresses in environment. Nevertheless, the defense mechanism in microalgae remains poorly understood. In this study, physiological and molecular methods were performed to analyze the defense responses in green alga *Chlamydomonas reinhardtii*. It was speculated that the defense responses might mainly be due to the regulation of hormone signaling, indicating its potential role in alleviating the Pb toxicity besides other physiological and molecular defense responses like decrease in growth rate, chlorophyll content and photosynthesis efficiency, intensification of antioxidative mechanisms, regulation of transcription factors, trace elements chelation, and sequestration into vacuole via trace elements transporters. The sole differentially expressed ATP-binding cassette (ABC) transporters indicated that ABC transporters might play a very important role in the transport and relocation of Pb in *C. reinhardtii*. Additionally, our data provide the required knowledge for future investigations regarding Pb toxicity and defense mechanisms in algae, and detection of trace elements pollution in environment.

## Introduction

Trace elements pollution has attracted the global attentions due to their adverse effects, particularly the developing world which has witnessed rapid development of industry, and opted for modern agricultural technologies (Kavamura and Esposito, 2011; Miransari, 2011; Singh et al., 2016). Trace elements are classified into two different categories, (i) essential trace elements with a known physiological function, such as Cu, Zn, Fe, Mn, Mo, Ni, and Co, and (ii) non-essential trace elements without a known physiological function, such as Hg, Cr, Al, and Pb, etc. (Singh et al., 2016; Du et al., 2018). Suitable amount of these essential trace elements play number of important roles in physiological and biochemical processes of living organisms such as the biosynthesis of pigments, the efficiency of photosynthesis, the growth and development, the synthesis of DNA and other metabolites, and the modification of proteins (Jalmi et al., 2018). However, excess amount of essential trace elements or very low concentrations of non-essential trace elements can cause lethal toxicity to the living organisms (Hayat et al., 2012; Gill et al., 2013; Jalmi et al., 2018). It have been demonstrated that non-essential trace elements inhibit the growth of plants, lead to oxidative stress by the accumulation of reactive oxygen species at the cellular level, and damage macromolecules such as lipids, proteins and nucleic acids in cells (Hall, 2002; Sharma and Dietz, 2009; Hossain and Komatsu, 2013). Cd was previously thought to be a non-essential trace element, but recently, it was reported that Cd could be a cofactor in metalloenzymes (e.g., carbonic anhydrases) and decided the activity of total metalloenzymes in algae (Jensen et al., 2020).

Among all non-essential trace elements, lead (Pb), is one of the most toxic trace elements affecting the environment and has become worldwide concerned including China. Zhuang et al. (2018) reported 29.60 mg/kg the average concentration of Pb in the sediment of northern Jiaozhou Bay and 221 mg/kg Pb in the Beijiang River of Pearl River Delta, China, which significantly higher than the standard limit (i.e., 30.2 mg/kg) proposed by C.C.M.E. (Canadian Council of Ministers of the Environment) (Canadian Water Quality Guidelines, 1999; Zhao et al., 2017). The high concentration of Pb have been proved to have adverse effects on plants, algae and animals. For example, high concentration of Pb resulted in oxidative stress, DNA damage, the inhibition of seed germination and the decrease in growth of algae (i.e., *Chlamydomonas reinhardtii*) and plants [i.e., radish (*Raphanus sativus* L.)] (Szivák et al., 2009; Wang et al., 2013). Our previous study of Pb stress in alga *Gracilaria lemaneiformis* demonstrated its significant decrease in the growth, photosynthesis, and expressional proteins (Du et al., 2018). Therefore, it is necessary to study Pb stress in model microalgae in-depth, in order to generate a predictable model to assess impact of trace element in general and Pb toxicity in particular in organisms.

*Chlamydomonas reinhardtii*, a unicellular green alga, is one of the most important model organisms for studies under different biotic and abiotic stresses. Previously, considerable efforts have been put forward for investigating the responses of *C. reinhardtii* under Pb stress, which mainly focused on the role of phytochelatin in Pb detoxification (Scheidegger et al., 2011a, b), its bioaccumulation and biosorption (Chen et al., 2010; Flouty and Estephane, 2012; Stewart et al., 2015) and its transportation within cells (Sánchez-Marín et al., 2014). However, little is known about the defense mechanism of *C. reinhardtii* and its responses to Pb stress, particularly at the molecular level, which includes hormone signaling and transcription regulation of genes. Therefore, we used *C. reinhardtii*, a freshwater model microalga, to further investigate the physiological and molecular responses of algae on exposure to Pb stress. By doing so, following key questions were addressed (i) What physiological responses *C. reinhardtii* demonstrated under lead stress? (ii) What genes were regulated (i.e., transcription regulation) when algae were exposed to Pb stress? In order to find the possible answer of question – (i), growth rate, pigment content, and the activity of antioxidant enzymes were measured at physiological level. While, next-generation sequencing technology was employed to characterize the transcription regulation of genes to answer question – (ii). Additionally, expression profiling of some differentially regulated genes was validated by qRT-PCR. This is first comprehensive study of physiological and molecular responses (e.g., hormone signaling, transcription factors, trace element chelators and transporters) to Pb stress in *C. reinhardtii*, and our findings provide solid and reliable foundation for further in-depth study of impact of Pb toxicity and its detoxification/sequestration in algae and in other organisms and Pb detection/removal from environment.

## Materials and Methods

### Algae Culture and Treatments

The Freshwater Algae Culture Collection [Institute of Hydrobiology, FACHB (Wuhan, China)] provided *C. reinhardtii* (CC-503 cw903 mt^+^) used in this study. The cells were pre-cultured in the tris-acetate-phosphate (TAP) medium in an incubation chamber at 22°C, 12 L:12 D photoperiod, and 80 mmol photons m^–2^ s^–1^ light intensity by rotary shaker (100 rpm) (Hutner, 1990). Cells during mid-exponential growth were diluted to 1.0 × 10^5^ cells mL^–1^ with fresh medium and transferred to modified TAP medium (Kopittke et al., 2008). Na_2_-glycerol-2-phosphate (Gly-2-P) was added at a concentration of 2.7 mM.

Modified TAP medium was prepared in 10^–2^ M 2-(*N*-morpholino) ethane sulfonate (MES, sodium salt, Sigma) medium. To minimize the potential effect of carbonate complexes, the solutions were buffered to pH 6 (Hassler et al., 2004). Pb(NO_3_)_2_ stock solutions were prepared using analytical grade chemicals and were diluted to 0, 3, and 80 μmol.L^–1^ in ultrapure water, respectively. Nitrate was selected as the counter ion due to its low possibility to form trace element complex. Concentrated solutions of nitric acid were used for pH adjustment. Under these conditions, more than 96% of free Pb^2+^ trace element represented in medium in the absence of added ligand.

### Basic Parameters Analysis

#### Cell Density

Number of cells were measured using an electronic particle counter (Orifice, 50 μm; Multisizer II; Beckham Coulter, Fullerton, CA, United States) (Zheng et al., 2013).

Measurement of chlorophyll content: cells were collected by centrifugation at 3,700 *g* for 4 min. Chlorophyll was extracted in 80% methanol, and quantified by measuring absorbance at 650 and 665 nm (Juneau et al., 2005).

Determination of chlorophyll fluorescence parameters: A portable pulse amplitude modulated fluorometer Water-PAM (Walz, Effeltrich, Germany) was used to determine the chlorophyll fluorescence parameters. Prior to the measurements, The PSII electron transport chain was completely oxidized by putting the cells of *C. reinhardtii* in the dark for 10–15 min. Subsequently, triplicate of the maximal photochemical efficiency of PSII (*F*_v_/*F*_m_) was read on the Water-PAM by applying three saturation pulses (Samadani et al., 2017).

#### Enzymatic Assays

Algal cells were harvested by centrifugation at 3,700 *g* for 10 min at 4°C, washed with PBS buffer (three time). Finally, cells were resuspended in 1 mL of 0.1 M sodium phosphate buffer (PH 7.0) and were ruptured using ultrasonic homogenizer. The homogenate was then centrifuged at 13,523 *g* for 20 min at 4°C and the supernatant was considered as cell free enzyme extracts. Enzymatic assays were conducted by following the manufacturer’s instruction of commercially available kits (Peroxidase Assay Kit, Superoxide Dismutase Assay Kit, and Catalase Assay Kit), malondialdehyde (MDA) was measured by Malondialdehyde assay kit (Nanjing Jiancheng Bioengineering Institute, Nanjing, China).

All the data from triplicate experiments were shown as mean ± standard deviation (SD). Data were analyzed using SPSS 20.0 software with one-way ANOVA (Khan et al., 2019). The statistical significance was considered at ^∗^*P* < 0.05 or ^∗∗^*P* < 0.01. Data were represented as mean with different letters representing extremely significant differences in mean value among different treatments. All figures were generated by Origin 8.0.

### Transcriptome Analysis

#### RNA Extraction and Sequencing

RNA was extracted from algal cells on the third day of lead stresses using the RNAprep pure Plant Kit [Tiangen Biotech(Beijing) Co., Ltd., Beijing, China] and measured using NanoDrop 2000 (Thermo). RNA integrity was assessed using the RNA Nano 6000 Assay Kit of the Agilent Bioanalyzer 2100 system (Agilent Technologies, CA, United States). cDNA synthesis was performed using random primers [cDNA synthesis kit, Tiangen Biotech(Beijing) Co., Ltd.]. NEBNext UltraTM RNA Library Prep Kit for Illumina (NEB, United States) was used to generate to sequence libraries. Sequence reads are available by the NCBI sequence read archives [SRR10269729 (control), SRR10269730 (control), SRR10269727 (low lead stress), SRR10269728 (low lead stress), SRR10269731 (high lead stress), and SRR10269732 (high lead stress)].

#### Construction of Transcriptomic Library

The library construction of transcriptome and bioinformatics analysis were conducted as previously described. Briefly, bioinformatics analysis of RNA sequences raw reads was transferred to clean reads by trimming adapter sequences, removing poly-N containing reads as well as filtering low-quality reads via proprietary procedures (20 *q*-scores for 50% nucleotides per read). Transcriptome assembly was performed by trinity platform with the two sets of sequence reads as the input (Haas et al., 2013). Prior to differential gene expression analysis, for each sequenced library, the read counts were adjusted using edgeR program package by one scaling normalized factor. Differential expression analysis of two samples was performed by the DEGseq (2010) R package. Genes with |log2 fold change| ≥ 1 and FDR < 0.01 (adjusted *P*-value, determined by the Benjamini and Hochberg multiple-testing correction implemented in the ‘p.adjust’ method of *R*) were defined as DEGs. The value of FPKM was the average of two biological replicated experiments.

#### Gene Functional Annotation

Based on the following database: Nr (NCBI non-redundant protein sequences),^1^ Pfam (Protein family),^2^ KOG/COG (Clusters of Orthologous Groups of proteins),^3^ (see Footnote 3) Swiss-Prot (A manually annotated and reviewed protein sequence database),^4^ KO (KEGG Ortholog database),^5^ GO (Gene Ontology),^6^ gene function was annotated. The whole KOG/COG and Pfam database were used for the annotation of gene function. While the algal and plants database of Nr, Swiss-Prot, KEGG and GO were used for the annotation of gene function. E value was −e 1e-5.

#### Quantification of Gene Expression Levels

Quantification of gene expression levels were estimated by fragments per kilobase of transcript per million fragments mapped. The formula is shown as follow:

$$\text{FPKM}=\frac{\mathrm{cDNA}\,\mathrm{Fragments}}{\mathrm{Mapped}\,\mathrm{Fragments}\,\left(\text{Millions}\right)\times \mathrm{Trascript}\,\mathrm{Length}\,\left(\text{kb}\right)}$$

#### Validation of DEG Expression With Quantitative Real-Time PCR (qRT-PCR)

Reverse transcription quantitative PCR (qRT-PCR) was performed as previously report (Liu et al., 2019) to determine the transcriptional levels of DEGs. All qPCR reactions were performed using Quant qRT-PCR kit [Tiangen Biotech(Beijing) Co., Ltd., Beijing, China]. The primers used for qRT-PCR were listed in Supplementary Table S1. The standard curve of qRT-PCR was determined with six-serial dilutions of first-chain cDNA template. The comparative threshold (2^–ΔΔ^*^*C*^*^*t*^) method was used to assess the relative expression levels. For every primer pair, a standard curve was performed to analyze the amplification efficiency and to identify potential primer dimers.

#### Gene Ontology (GO) Enrichment Analysis

Gene Ontology enrichment analysis of the DEGs was carried out through the GOseq R packages based on Wallenius non-central hyper- geometric distribution, which can be used to adjust gene length bias in DEGs (Chen et al., 2015).

#### KEGG Pathway Enrichment Analysis

KEGG is a database resource to understand high-level functions and utilities of the biological system from molecular-level information, especially, large-scale molecular datasets generated by genome sequencing and other high throughput experimental technologies (see Footnote 6). KOBAS software was used to determine the statistical enrichment of DEGs in KEGG pathway (Chen et al., 2015).

## Results

### Physiological Analysis of *C. reinhardtii* Under the Lead Treatment

**FIGURE 1:**
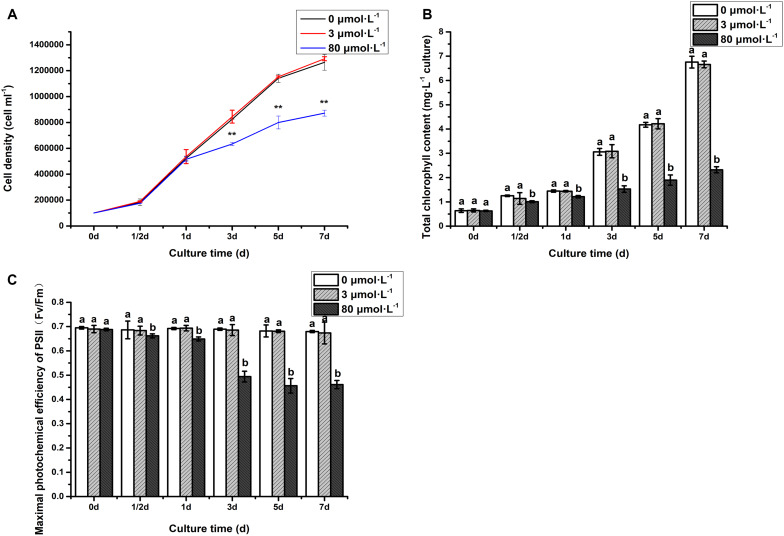
Cell density **(A)**, chlorophyll content **(B)**, and *F*_v_/*F*_m_ **(C)** of algae under the lead treatment. Data were represented as mean with **P < 0.01. Different letters representing extremely significant differences in mean value between different treatments. Error bars indicate SD for three biological replicates.

To investigate the response of *C. reinhardtii* under the lead treatment, some key physiological parameters were measured throughout the experiment (Figure 1). It can be seen that the algal cell density under the lead treatments (3 and 80 μmol.L^–1^) was consistent with that of control on the first day (Figure 1A). However, compared to control and 3 μmol.L^–1^ lead treatment, hereafter referred as low concentration, the algal cell density was significantly declined after 3 days when treated with 80 μmol.L^–1^ lead (*P* < 0.01). The cell density was decreased in continuously fashion, i.e., 23.20, 29.90, and 31.03% on the 3rd, 5th, and 7th days, respectively. The both total chlorophyll content and *F*_v_/*F*_m_ of algae under 80 μmol.L^–1^ lead treatment were significantly declined on the 1/2 day (*P* < 0.05) with extremely significant declined demonstration on the 1st, 3rd, 5th, and 7th days (*P* < 0.01) (Figure 1). Briefly, the total chlorophyll content was decreased 19.61, 16.05, 50.07, 54.58, and 65.63% on 1/2 day, 1st, 3rd, 5th, and 7th day, respectively compared to the control and low lead treatment (Figure 1B). While the *F*_v_/*F*_m_ was declined 3.54, 6.21, 28.28, 33.08, and 32.12% on 1/2 day, 1st, 3rd, 5th and 7th day, respectively, compared to the control and low lead treatment, i.e., 3 μmol.L^–1^ (Figure 1C).

**FIGURE 2:**
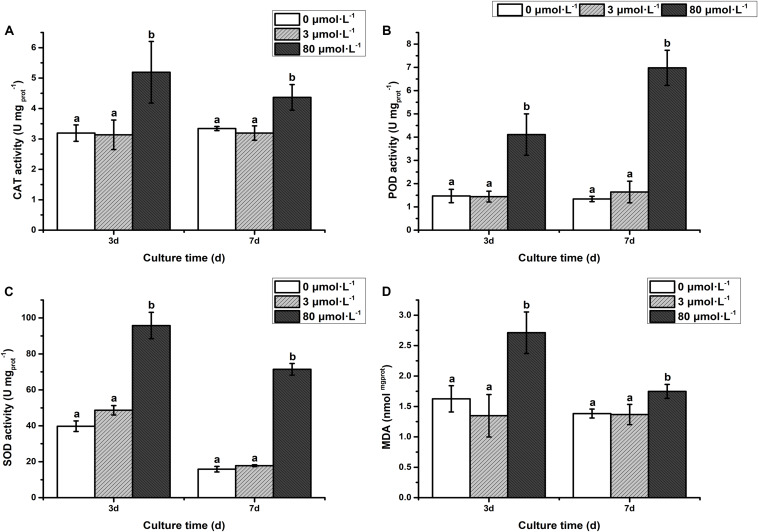
**(A)** Catalase activity, **(B)** Peroxidase activity, **(C)** Superoxide Dismutase activity, **(D)** Malondialdehyde content. The activity of antioxidant enzymes and MDA content of algae under the lead treatment. Different letters representing extremely significant differences in mean value between different treatments. Error bars indicate SD for three biological replicates.

As depicted in Figure 2, the activity of antioxidant enzymes [catalase (CAT), peroxide dismutase (POD), and superoxide dismutase (SOD)] and malondialdehyde (MDA) in the low lead treatment were not significantly different from that of the control (*P* > 0.05). However, the CAT activity was significantly increased on the 3rd day with extremely remarkably increased on the 7th day of treatment with high lead compared to control and low lead treatment (Figure 2A). The POD activity was extremely higher than control and low lead treatment on the 3rd and 7th days of experiment (*P* < 0.01) (Figure 2B). Furthermore, it was observed that the POD activity under the high lead treatment was increased 1.8- and 5.2-fold compared with control group, respectively. Similarly, the SOD was also extremely prominently higher than that of control and low lead treatment on the 3rd and 7th days (*P* < 0.01) (Figure 2C). The SOD activity under the high lead treatment was raised 1.4- and 4.5-fold compared with control group, respectively. Moreover, compared to control and low lead treatment highly significant increased MDA content on the 3rd day less but still significantly increased on the 7th day was also observed (Figure 2D).

### Transcriptome Analysis of *C. reinhardtii* Under the Lead Treatment

#### General Transcriptome Analysis of *C. reinhardtii* Response to Lead Stress

By sequencing the transcriptome of *C. reinhardtii*, fifty-eight to sixty-eight million clean reads of each sample were achieved after filtering, with more than 93% bases showing quality greater than Q30. While the GC percentage was around 62% (Table 1). Based on the comparison, variable splicing prediction analysis, gene structure optimization analysis and the discovery of new genes were conducted. 482 new genes were discovered, 353 of which were functionally annotated.

**TABLE 1:** Illumina sequencing statistics.

Sample	Clean reads	Mapped reads	Uniq mapped reads	GC content	% ≥Q30
0 μmol ⋅ L^–1^	58,002,664	51,774,827 (89.26%)	49,728,827 (85.74%)	61.80%	93.46%
0 μmol ⋅ L^–1^	68,496,900	62,133,377 (90.71%)	59,159,451 (86.37%)	61.77%	94.11%
3 μmol ⋅ L^–1^	61,544,320	55,391,603 (90.00%)	53,164,273 (86.38%)	61.49%	93.81%
3 μmol ⋅ L^–1^	65,010,892	57,638,918 (88.66%)	55,159,746 (84.85%)	62.10%	93.59%
80 μmol ⋅ L^–1^	59,878,480	54,229,052 (90.57%)	50,313,271 (84.03%)	62.66%	93.81%
80 μmol ⋅ L^–1^	68,896,800	63,775,217 (92.57%)	59,876,803 (86.91%)	64.10%	94.50%

#### Differentially Expressed Genes (DEGs) Affected by the Lead Treatment

**FIGURE 3:**
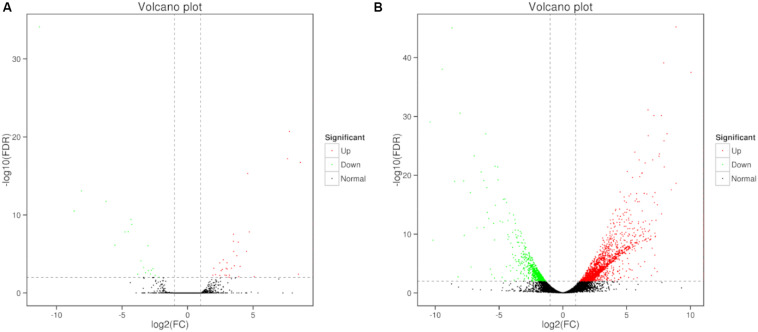
Volcano plots of DEGs in *C. reinhardtii*. Panel **(A)** was the comparison of DEGs in 0 vs. 3 μmol.L^–1^. Panel **(B)** was the comparison of DEGs in 0 vs. 80 μmol.L^–1^.

Differential expression of genes was also analyzed. Compare to the control, large number of differentially expressed genes (DEGs) were identified in 0, 3, and 80 μmol ⋅ L^–1^ lead treatment. DEGs were annotated by matching against non-redundant protein sequence (Nr), Swiss-Prot, Kyoto Encyclopedia of Genes and Genomes (KEGG), Clusters of Orthologous Groups of proteins (COG), eukaryotic Orthologous Groups (KOG), Gene ontology (GO), and Pfam database. The number of *C. reinhardtii* DEGs based on different database was shown in Table 2. Total, 66 (40 up-regulated and 26 down-regulation) DEGs were identified in the comparison of 0 vs. 3, and 2,630 (1,866 up-regulated and 764 down-regulation) DEGs were identified in the comparison of 0 vs. 80. The volcano plots for DEGs of each comparison group were shown in Figure 3.

**TABLE 2:** The number of *C. reinhardtii* DEGs based on different database.

DEG Set	Total genes	Total annotated genes	COG	GO	KEGG	KOG	NR	Pfam	Swiss-Prot
0 vs. 3	66	58	11	52	3	24	58	36	49
0 vs. 80	2630	2147	401	1740	178	664	2146	1161	1800

*COG, Clusters of Orthologous Groups of proteins; GO, Gene ontology; KEGG, Kyoto Encyclopedia of Genes and Genomes; KOG, eukaryotic Orthologous Groups; NR, NCBI non-redundant nucleotide sequences; Pfam, Protein family; Swiss-Prot, A manually annotated and reviewed protein sequence database.*

#### Annotations of Differentially Expressed Genes (DEGs)

**FIGURE 4:**
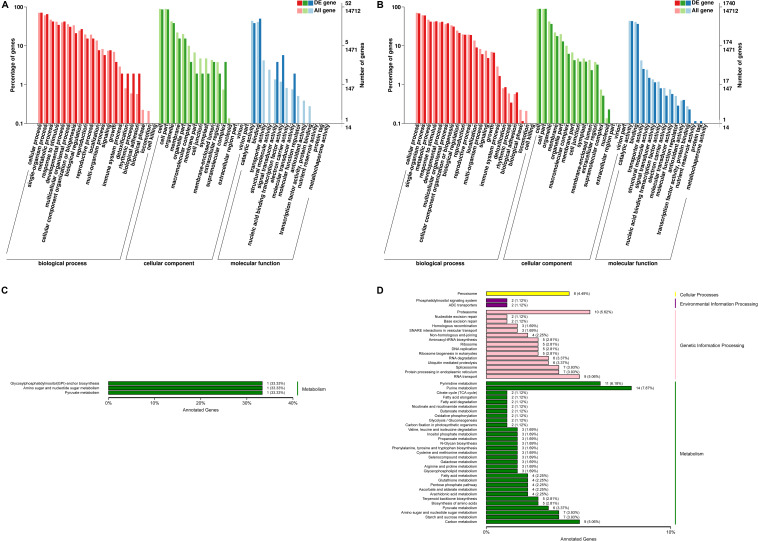
Functional annotation of differentially expressed genes from *C. reinhardtii*. **(A,B)** Gene Ontology (GO) annotation based on three main catteries: “Biological Process,” “Cellular Component,” and “Molecular Function.” **(C,D)** Kyoto Encyclopedia of Genes and Genomes (KEGG) pathways classification of the annotated differentially expressed genes based on four main catteries: “Genetic Information Processing,” “Environmental Information Processing,” “Cellular Processes,” and “Metabolism.” Panel **(A,C)** was the functional annotation of DEGs in 0 vs. 3 μmol.L^–1^. Panel **(B,D)** was the functional annotation of DEGs in 0 vs. 80 μmol.L^–1^.

To evaluate the *C. reinhardtii* transcriptome at the functional level, an analysis was carried out to determine the distribution of the abundance of the GO terms annotated to biological process, cellular component and molecular function (Figures 4A,B). The analysis of the biological processes revealed the particular abundance of transcripts involved in cellular process, single-organism process, metabolic process, response to stimulus, developmental process, multicellular organization or biogenesis, reproduction and localization. It was shown that 35 genes putatively related with hormones were observed from signal biological process under the high lead stress (see Supplementary Material 1), no genes putatively related with calcium and MAPK signals were found from in this GO category. As for cellular component, most of which were related to function in the cell, cell part, organelle, membrane, organelle part, macromolecular complex, cell junction and symplast. Fourteen significant terms were identified in the molecular function. These were transcription factor activity, protein binding, nucleic acid binding transcription factor activity, catalytic activity, signal transducer activity, structural molecule activity, transporter activity, binding, electron carrier activity, antioxidant activity, protein tag, nutrient reservoir activity, molecular transducer activity, molecular function regulator. In signal transducer activity, four genes putatively related to hormones were observed, but genes putatively involved in calcium and MAPK signals were not found from signal transducer activity (see Supplementary Material 1). Compared to the low lead treatment the most markedly distinction under the high lead treatments is that more differentially expressed genes were related with molecular function, including transporter activity, structural molecule activity, electron carrier activity, molecular function regulator, transcription factor activity, protein binding, antioxidant activity, nutrient reservoir activity and protein tag. The details of the GO classification of the above comparisons were presented in Supplementary Material 2.

Using the KEGG pathway classification 178 DEGs were annotated and assigned to 87 metabolic pathways. The results showed one up-regulated and two down-regulated KEGG pathways in the transcriptome analysis of algae under the 0 vs. 3 μmol.L^–1^ lead treatment. At the same time the results revealed 141 up-regulated and 37 down-regulated KEGG pathways under the 0 vs. 80 μmol.L^–1^ lead treatment. These unigenes annotated using database were classified into four categories, including cellular processes, environmental information processing, genetic information processing and metabolism. Obviously, differentially expressed genes on the low lead treatment were only focused on the three pathways of metabolism, including Glycosyphosphatidylinositol (GPI)-anchor biosynthesis, amino sugar and nucleotide sugar metabolism, and pyruvate metabolism (Figure 4C). However, the up- and down-regulated genes on the high lead treatment were involved in all four KEGG categories, including cellular processes, environmental information processing, genetic information processing and metabolism (Figure 4D). The top 10 pathways were purine metabolism, pyrimidine metabolism, proteasome, RNA transport, carbon metabolism, peroxisome, spliceosome, protein processing in endoplasmic reticulum, amino sugar and nucleotide sugar metabolism and starch and sucrose metabolism. The details of the KEGG classification of the above comparisons were presented in Supplementary Material 3.

Additionally, genes involved in potential signaling pathway and its ultimate responses were analyzed. The transcriptome data of *C. reinhardtii* showed that a prominent large proportion of DEGs was putatively involved in hormones signaling under the lead treatment (Table 3). In total, 67 DEGs putatively related to plant growth hormones were identified. They were classified into seven groups, including gibberellin (GA), cytokinin (CK), auxin, abscisic acid (ABA), ethylene, brassinosteroids (BR), and salicylic acid (SA) (Supplementary Tables S2–S8). The results showed that compared with the expression of genes in control group one gene putatively response to salicylic acid, one gene putatively response to brassinosteroid, one gene putatively response to auxin, two genes putatively response to abscisic acid, one gene putatively response to cytokinin and two genes putatively response to ethylene were up-regulated under the low lead treatment. No down-regulated gene was found under the low lead treatment. However, 67 genes were found to be differently expressed under the high lead treatment. Among them genes response to GA, CK, and BR hormones were all up-regulated under the high lead treatment in *C. reinhardtii*.

**TABLE 3:** The number of DEGs based on three signaling networks.

	Hormone signaling		Calcium signaling		MAPK signaling	
	0 vs. 3	0 vs. 80	0 vs. 3	0 vs. 80	0 vs. 3	0 vs. 80
Number of up-regulated genes	3	44	0	0	0	0
Number of down-regulated genes	0	25	0	0	0	0

Downstream responses majorly include the regulation of transcription factors, trace element chelators and transporters. In this study, 20 genes putatively associated with transcription factors were annotated based on NCBI or Swiss-Prot database (Supplementary Table S9). These genes encode C_2_H_2_ (C_2_H_2_-type zinc finger transcription factor), AP2 (activator protein 2), MYB (myeloblastosis), bHLH (basic-helix-loop-helix), bZIP (basic region leucine ZIPper), SBP (SQUAMOSA promoter binding proteins), YABBY, GATA, HB (homeobox)-other, and MYB (myeloblastosis)-related transcription factors. It was shown that only one up-regulated gene and one down-regulated gene under the low lead treatment compared to the control. They were annotated to encode transcription factor associated with GATA. All the other genes were not differentially expressed under the low lead treatment. However, all genes encoding C_2_H_2_, AP2, MYB, bHLH, YABBY transcription factors were upregulation under the high lead treatment compared to that in control. Genes encoding bZIP, SBP and HB-other transcription factors were down-regulated. Among all genes encoding trace element chelators, only the genes encoding proteins putatively involved in phytochelatin and glutathione were found to be differentially expressed on the transcriptome data of *C. reinhardtii* under the high lead treatment. Among all annotated phytochelatin genes, only one gene was differentially expressed under the high lead treatment. Annotation based on NCBI or KEGG-pathway database showed that differentially expressed genes putatively related to glutathione metabolism were not observed in control and low lead treatment (Supplementary Table S10) but observed under the high lead treatment including three up-regulated genes and one down-regulated gene. Transcriptome data of *C. reinhardtii* under the lead stress revealed that ABC transporters were the sole differentially expressed genes among all trace element-transporters. Annotation-based on NCBI or Swiss-Prot database showed that differentially expressed genes encoding putatively ABC transporters were not observed between control and low lead treatment (Supplementary Table S11). While, 10 differentially expressed genes comprised of 4 up-regulated and 6 down-regulated genes were found in control and high lead treatment.

#### The Results of Real-Time Quantitative PCR

Genes expression of ABC-transporter, transcription factor, peroxisome activity and glutathione metabolism validate the sequencing results obtained by RNA-seq. Overall, the expression levels of all the genes were similar in the results of RNA-seq and qRT-PCR (Table 4). The regression analysis with R values (0.7212 and 0.8155, respectively) under the lead stresses was shown in Supplementary Figures S1, S2. The levels of gene expression have some small differences between the RNA-seq and qRT-PCR measurements. This difference might be affected by different measurement methods.

**TABLE 4:** The results of qRT-PCR.

Gene ID	Annotation	Group	RNA-seq	qPCR
Cre12.g540650	ABC-2 type transporter	0 vs. 3	−0.24759	−0.12867
		0 vs. 80	−1.86927	−2.06236
Cre12.g530900	ABC-2 type transporter	0 vs. 3	−0.13803	−0.22134
		0 vs. 80	−2.37844	−3.33791
Cre01.g009575	AP2 domain	0 vs. 3	0.959535	0.72746
		0 vs. 80	3.52753	2.82337
Cre07.g353300	Solute carrier family 25	0 vs. 3	−0.44481	−0.62
		0 vs. 80	−2.62199	−2.43061
Cre06.g297900	Protein Mpv17	0 vs. 3	NA	−0.39643
		0 vs. 80	3.84121	3.57696
Cre12.g559800	Glutathione *S*-transferase	0 vs. 3	0.104398	0.066
		0 vs. 80	8.872299	10.1766
Cre17.g703176	Superoxide dismutase	0 vs. 3	NA	0.11321
		0 vs. 80	Inf	5.55531
newGene_730	Thioredoxin m	0 vs. 3	−0.04252	−0.29183
		0 vs. 80	−1.41185	−2.01768
Cre08.g370650	6-phospho- gluconolactonase	0 vs. 3	NA	0.36086
		0 vs. 80	6.21393	5.20633
Cre08.g378850	Ribose-phosphate pyrophosphokinase	0 vs. 3	−0.13191	−0.11702
		0 vs. 80	−1.76091	−2.07
Cre16.g675650	Malonate-semialdehyde dehydrogenase	0 vs. 3	−0.46	−0.4475
		0 vs. 80	1.45515	1.54112

*NA, not analyzed; Inf, infinity. RNA-seq and qPCR is the average result of different replicates.*

## Discussion

Physiological responses showed that the growth rate (cell density), chlorophyll content and photosynthesis efficiency (*F*_v_/*F*_m_) were inhibited under the high lead stress in *C. reinhardtii*. Our finding in terms of decrease in growth, chlorophyll content and photosynthesis efficiency under Pb stress are consistent with previous reports such as in *Chlorella* and cabbage etc. (Pandey and Sharma, 2002; Dao and Beardall, 2016). The Pb concentration of inhibiting algal growth in our *C. reinhardtii* CC-503 cw903 mt^+^ was much higher than the concentration reported in *C. reinhardtii* CCAP 11/32B (De Schamphelaere et al., 2014), this might be because both *C. reinhardtii* are different species. The significant decrease in growth rate, chlorophyll content and photosynthesis efficiency were suggested to due to the impairment of photosynthesis in microalga. Moreover, previous reports indicated that ROS production inhibited the growth (Bertrand and Poirier, 2005; Stoiber et al., 2013). This might be the possible explanation of growth rate inhibition under the high Pb treatment in this study. Since, it has been known that Pb damage the ultrastructure of chloroplasts (Qiao et al., 2013) therefore, inhibition of chlorophyll synthesis and photosynthesis efficiency were observed in *C. reinhardtii*. The maximum quantum yield of PSII (*F*_v_/*F*_m_) is a sensitive indicator of photosynthesis efficiency. The value is maintained at 0.6 to 0.65 for healthy algae (Maxwell and Johnson, 2000; Parkhill et al., 2001). However, *F*_v_/*F*_m_ was found lower than 0.6 from the 3rd day onwards, indicating the low photosynthesis and unhealthy state of alga which ultimately resulted in significantly less cell density on the 3rd day. Reactive oxygen species were commonly induced under the pressure of trace elements including Pb (Szivák et al., 2009). And it was also known that SOD, POD, and MDA play an important roles in antioxidative defense against Pb-induced oxidative stress (Gechev et al., 2006; Dao and Beardall, 2016). Therefore, it was speculated that CAT, SOD, and POD activity were enhanced, MDA content was increased to alleviate Pb-induced oxidative stress in *C. reinhardtii*.

In addition to physiological level, the transcriptome level of *C. reinhardtii* was also analyzed and discussed under the Pb treatment. GO terms annotation suggested that compared to biological process and cellular component, molecular function played a more important role in algal adaptation to lead stress. Several genes putatively related with hormones were enriched in signaling of biological process and signal transducer activity of molecular function, while genes putatively involved in calcium signaling and MAPK signaling were not differentially expressed or enriched in signaling of biological process and signal transducer activity. This could be explained by the importance of genes putatively related with hormones under the trace element stresses. DEGs putatively involved in electron carrier activity and antioxidant activity explained that the photochemical efficiency, pigment content, the activity of antioxidant enzymes and the MDA content of algae were significantly affected under the high lead stress. KEGG pathway classification showed that more pathways were activated in response to the high lead stress on microalgae. This indicated that compared to the metabolism pathway in the low lead stress more pathways involved in genetic information processing, environmental information processing and cellular processes were activated to defense the stress of high lead. Among these activated pathways, differentially expressed genes putatively involved in peroxisome pathway were observed, which might explain the enhanced activity of antioxidant enzymes. This hypothesis was also supported by a previous paper showing that the action of antioxidant enzymes (e.g., SOD, CAT, ascorbate peroxidase, and glutathione reductase) was to resist oxidative stress (Matilla-Vázquez and Matilla, 2012).

**FIGURE 5:**
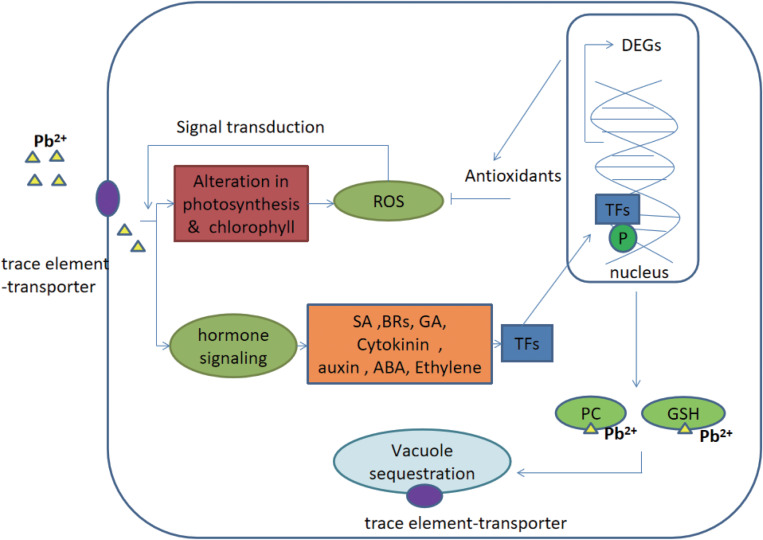
Potential hormone signaling pathway and its ultimate response in *C. reinhardtii* under the lead treatment. Gas, gibberellins; CKs, cytokinins auxins; ABA, abscisic acid, ethylene; BR, brassinosteroids; SA, salicylic acid; PC, phytochelatins; GSH, glutathione; TF, transcription factors; ROS, reactive oxygen species; P, phosphorylated; and DEGs, differentially expressed genes. The figure was modified from Jalmi et al. (2018).

Generally, signaling cascades were thought to be an important contribution during the defense responses of plants (Jalmi et al., 2018). The signal was achieved from the upstream receptors and transmitted into the nucleus. Afterward, different stresses-responsive genes cooperatively and/or separately were regulated by transcription factors and thus, gene networks were constituted. Finally, genes were differentially expressed to help plants to adapt to various environmental stresses (Singh et al., 2016). There are many signaling networks working in the stress of trace elements, majorly including hormone signaling, calcium signaling and MAPK signaling (Rao et al., 2011; Sinha et al., 2011; Chen et al., 2014; Steinhorst and Kudla, 2014). Go annotation showed that compared to genes putatively related with calcium and MAPK signals the genes putatively related to hormone signaling were only differentially expressed and annotated in this study. Therefore, it was speculated that hormone signaling was an important signaling network in *C. reinhardtii* under the lead treatment. Addition to the synthesis of hormones signaling molecules, trace elements stress-related proteins (e.g., trace element chelators and transporters) were also accumulated in cells (Mendoza-Cózatl et al., 2011). These proteins were putatively involved in chelating and sequestering trace elements to vacuoles (Hasan et al., 2017). Therefore, hormone signaling regulation network, and its downstream responses (i.e., the regulation of transcription factors, trace element chelators and transporters) were further discussed in this study (Figure 5).

It was known that different plant hormones play an important role in trace element stress response (Cao et al., 2009; Vitti et al., 2013; Chen et al., 2014; Zhao et al., 2014). In this study, 67 genes putatively related to plant growth hormones were differently expressed under the high lead treatment, indicating a complex regulation network. Genes putatively encoding GA, CK, and BR hormones were all up-regulated under the high lead treatment, suggesting potential important role of GA, CK, and BR in algal defense responses. It was reported that regulation of CAT and POD activities or the overexpression of genes related to GA hormone could alleviate detrimental effects of different trace elements (e.g., Ni, Cr, Zn, Cd, and Pb) on higher plants (Hamayun et al., 2010; Gangwar et al., 2011; Zhu et al., 2012). Accumulation of CK hormone in plants help them to mitigate the trace element induced toxicity (Jalmi et al., 2018). Furthermore, the destruction of photosynthetic pigment and chloroplast membrane could be repaired by CK hormone to increase photosynthetic capacity (Piotrowska-Niczyporuk et al., 2012). Several studies revealed that BR could regulate the antioxidant defense system to mitigate the toxicity of trace elements (e.g., Cu, Pb, and Cd) in *Chlorella vulgaris* (Bajguz, 2010). Additionally, it was revealed that the jasmonic acid and the BR pathways had antagonistic action in infected higher plants (He et al., 2017). BR related Cre02.g076800 and Cre12.g511700 genes were analyzed under the methyl jasmonate treated *C. reinhardtii*, no differentially expressed Cre02.g076800 could be explained by that Cre02.g076800 was putatively involved in the BR pathway but not jasmonic acid pathway, while Cre12.g511700 gene was proposed to function under the treatment of phytohormone methyl jasmonate (Commault et al., 2019). Compared to the treatment of methyl jasmonate, these two genes were up-regulation in the Pb treated *C. reinhardtii*. Therefore, it was speculated that GA, CK, and BR hormones might play an important role in regulating the activity of antioxidant enzymes, pigment content, algal photosynthesis, and alleviating the Pb toxicity in *C. reinhardtii* under the high lead treatment.

In addition, some salicylic acid (SA), auxin, abscisic acid (ABA) and ethylene putatively related genes were also differentially expressed under the lead stress. Among six up-regulated and five down-regulated genes putatively involved in SA, Cre06.g275350 gene was up-regulation under the low and high lead stresses. In previous study, it was shown that Cre06.g275350 gene was putatively involved in the control of gene expression in *C. reinhardtii* (Formighieri et al., 2012). Therefore, it was proposed that its upregulation might regulate the gene expression during the SA pathway under the lead stress. While Cre06.g287000 gene was only upregulation under the high lead stress. The Cre06.g287000 was homologous with AT2G33380 in *Arabidopsis* and Cz16g16140, Cz09g31050, and Cz03g13150 in oleaginous alga *Chromochloris zofingiensis* involved in defense against stresses (Wang et al., 2019). Hence, it is reasonable to speculate that the upregulation of Cre06.g287000 might also play roles in *Chlamydomonas* defense against lead stress in this study. According to the previous paper, it was speculated that the auxin related Cre09.g394436 gene might participate in the polar transport of auxin and sodium in *C. reinhardtii* (Goodenough et al., 2019). Cre09.g392350 gene had the same response under the CO_2_ limitation and lead stress, both showing a slightly up-regulated expression (Arias et al., 2020). However, Cre03.g186950 expression was down-regulation under the salt stress and up-regulation under the lead stress in *C. reinhardtii* (Li et al., 2017). Both genes were annotated to response to abscisic acid, suggesting the response of gene expression might be dependent on different abiotic stresses. Cre10.g422900 gene was induced to be co-expressed with other genes in *C. reinhardtii* under the cold stress (Li et al., 2020). Anyway, the accurately function of these genes must be certificated by experimental methods.

Transcription factors play an important role in regulation of gene expression in many defense responses (e.g., trace elements responses) in plants (Yanhui et al., 2006). Previous studies documented that the overexpression of transcription factors in plants increased trace element tolerance (Cho et al., 2007; Yamaji et al., 2009; Long et al., 2010; Blanvillain et al., 2011; Gao et al., 2013). The overexpression of C_2_H_2_ increased the tolerance of plants to Al toxicity by regulating proton pump (Iuchi et al., 2007). Overexpressed AP2 protected plants against oxidative stress induced by trace elements through enhancing the production of APX, GR, and SOD (Tang et al., 2005). Previous studies have also demonstrated that the expression of AP2 and MYB were up-regulated on exposure to trace elements, mutation in MYB gene resulted in the increase of trace element sensitivity, the significant accumulation of H_2_O_2_ and decrease of CAT activity in higher plants (Van De Mortel et al., 2008; Ogawa et al., 2009; Hu et al., 2017). Overexpression of bHLH gene increased the sequestration of Cd in plants (Wu et al., 2012). These studies further indicated that the up-regulation of genes encoding C_2_H_2_, AP2, MYB, bHLH, and YABBY transcription factors in this study might have positively effects in the mechanism of Pb stress tolerance, through increasing the activity of CAT, POD, and SOD, enhancing the production of MDA and the sequestration of Pb. However, owing to some other transcription factors, including bZIP, SBP, and HB-other transcription factors were reported to involve in uptake and accumulation of trace elements, they might have negative effect on the regulation of Pb stress (Song et al., 2019).

Trace element chelators were also found under the trace elements stresses, including phytochelatins, metallothioneins, glutathiones, serine acetyltransferase, histone and cys-rich trace element binding peptides (Kumar et al., 2015; Jalmi et al., 2018). Only the genes putatively encoding phytochelatins and glutathiones were observed in DEGs of *C. reinhardtii*, indicating that chelators might have trace element and organism specificity. It has already been known that phytochelatin synthesis is important in regulating plant tolerance to trace elements (Clemens, 2006; Emamverdian et al., 2015). Only one annotated phytochelatin gene in this study was differentially expressed under the lead treatments. This might be explained by that the biosynthesis of PCs can be regulated at post-translational level in plants (Hasan et al., 2017).

It was known that the transport and relocation of trace elements in cells was carried out by different transporters localized in the plasma and vacuolar membranes. Many transporters were identified from plants and algae, such as zinc-iron permease, ATP-binding cassette (ABC)-transporters, natural resistant associated macrophage protein, cation diffusion facilitator and trace element transport ATPase (Park et al., 2012; Singh et al., 2016). In this study, putatively ABC transporters were the sole differentially expressed genes on the transcriptome data of *C. reinhardtii* under the lead treatments. Two differentially expressed genes putatively involved in ABC transporter pathway were enriched by KEGG analysis. This might be because trace element transporters have trace element and organism specificity. ABC transporters were special for the transport and relocation of Pb trace element in *C. reinhardtii*. ABC transporters have already been reported to be a major trace elements (e.g., Pb) transporters and play an vital role in vacuole sequestration and detoxification of trace elements in plants (Kim et al., 2007; Wang et al., 2017).

## Conclusion

Adverse effects of trace elements have compelled the scientists across the globe to come-up with realistic solution for its control. Microalgae are one of the most investigated organisms for trace element bioaccumulation potentials. However, existing knowledge regarding microalgae response against trace elements at molecular level is insufficient. Therefore, in this study, we aimed to fill this knowledge gap by investigating the responses of model microalga *C. reinhardtii* both at physiological and molecular levels under Pb stress.

Our data revealed that *C. reinhardtii* demonstrate physiological responses under Pb stress in species dependent manner. Moreover, it was speculated that hormone signaling pathway was important in defense mechanism of *C. reinhardtii* under Pb stress. Among all known trace element transporters, ABC transporter was found to be the sole differentially expressed gene in *C. reinhardtii*. It was indicated that ABC transporter played a very important role in the transport and relocation of Pb in *C. reinhardtii*. Anyway, the biological roles of hormone signaling pathway and ABC transporters in algae still need to be verified by further experiments. Our data is significant addition in the existing knowledge regarding the Pb toxicity and defense mechanism of microalgae. This model study would be helpful for future investigation and developing tools for trace element detection in both biological and non-biological systems.

## Data Availability Statement

The datasets presented in this study can be found in online repositories. The names of the repository/repositories and accession number(s) can be found in the article/Supplementary Material.

## Author Contributions

XL and HD designed the experiments and supervised the project. CZ, MA, XX, HJ, NH, KT, YY, and PL performed the experiments. XL, HD, CZ, and MA wrote the draft manuscript. All authors discussed the results and implications and commented on the manuscript at all stages.

## Conflict of Interest

The authors declare that the research was conducted in the absence of any commercial or financial relationships that could be construed as a potential conflict of interest.

## Footnotes

1 ftp://ftp.ncbi.nih.gov/blast/db/

2 http://pfam.xfam.org/

3 http://www.ncbi.nlm.nih.gov/COG/

4 http://www.uniprot.org/

5 http://www.genome.jp/kegg/

6 http://www.geneontology.org/
