# Supplementary material for: Impact of Pb on Chlamydomonas reinhardtii at Physiological and Transcriptional Levels
Source: Front Microbiol. 2020 Jun 26;11:1443. doi: 10.3389/fmicb.2020.01443 (PMC7333365; doi:10.3389/fmicb.2020.01443)
Supplement: Supplementary file 1 [file Data_Sheet_1.docx]

Supplementary Material 1

**Table S1**. Primers used for qRT-PCR

| Gene Name | Primers (5’-3’) |
| --- | --- |
| Cre12.g540650 | F: TTCGTGCTGCTGTGGAA  R: GTAGTGGTAGTTGAAGGTCTGCT |
| Cre12.g530900 | F: TCGCCTATCGGCAGAAA  R: CGTAATGGCTCCAGTGACA |
| Cre01.g009575 | F: CGTCAGACCAGCAGCAGTAT  R: CCCATAACGCCAGTAATCG |
| Cre17.g703176 | F: TAGCAGAAGCACGGACG  R: TGATGAGATGACGGGCTC |
| Cre07.g353300 | F: GCTGTTGGCTCGGTAATAG  R: AAGTAGACTGCCTGGCTGA |
| Cre06.g297900 | F: CAGTGGTGAAGCCCAAA  R: ACCTCCTGAGTTGCTGTCTC |
| Cre12.g559800 | F: TGAACGCACGCAAGAA  R: CGCTGAACGCAGTTGAA |
| Cre08.g370650 | F: GACGAGCAGCCAGATGA  R: CACCGCCAAGAAGAAGG |
| Cre08.g378850 | F: GTGAGCGAGGTGATGAA  R: CGGCGTTAGTGATTGTC |
| Cre16.g675650 | F: TTCTTCGGGCTGGTCAT  R: AGTTCTCCGTCGTTGAGTCC |
| newGene_730 | F: TTCAAGGGTGGCAAGAAG  R:TAGTCCCGTGTGCTACTGTG |

**Table S2.** Up- and down-regulated genes involved in brassinosteroid.

| Gene ID | Annotation |  | FPKM^c^ |  | DESeq_log2FC | |
| --- | --- | --- | --- | --- | --- | --- |
|  |  | 0 | 3 | 80 | 0vs3 | 0vs80 |
| Cre01.g019750 | GO:0009741 | 0.7446 | 0.8814 | 4.7228 | 0.2811 | 2.0322 |
| Cre02.g076800 | GO:0016126 GO:0016132 | 2.1542 | 8.7248 | 11.9056 | 2.2244 | 2.0460 |
| Cre05.g234656^b^ | GO:0009742 | 0.0309 | 0.0353 | 0.4634 | -- | 3.2850 |
| Cre07.g340900 | GO:0009741 | 2.2756 | 2.0966 | 12.7425 | -0.2072 | 1.8482 |
| Cre12.g501000 | GO:0016132 | 0.9134 | 0.0820 | 11.0842 | -- | 3.0304 |
| Cre12.g511700 | GO:0016132 | 2.2320 | 2.5219 | 39.5883 | 0.1953 | 3.4164 |
| Cre17.g735950^b^ | GO:0009742 | 0.4700 | 0.4383 | 2.6647 | -0.0474 | 1.8954 |

Abbreviations: a: genes related to hormone were enriched to signal transducer activity in molecular function. b: genes related to hormone were enriched to signaling biological process. c: the value of FPKM was the average of different experiments. --: DESeq_log2FC was extremely low. Red mark indicated that genes were up-regulated, green mark indicated that genes were down-regulated under the lead stress. Go annotation was carried out based on all plants’ database. GO:0009741: response to brassinosteroid; GO:0016126: sterol biosynthetic process; GO:0016132: brassinosteroid biosynthetic process; GO:0009742: brassinosteroid mediated signaling pathway.

**Table S3.** Up- and down-regulated genes involved in salicylic acid.

| Gene ID | Annotation | FPKM^c^ | | | DESeq_log2FC | |
| --- | --- | --- | --- | --- | --- | --- |
|  |  | 0 | 3 | 80 | 0vs3 | 0vs80 |
| Cre02.g102900 | GO:0009751 | 0.0527 | 0.0393 | 0.4004 | -- | 2.2902 |
| Cre06.g275350 | GO:0009751 | 0.1335 | 0.2645 | 0.9044 | 1.0158 | 2.1599 |
| Cre06.g287000 | GO:0009751 | 0.1106 | 0.1943 | 1.2971 | -- | 2.9491 |
| Cre06.g306601^b^ | GO:0009697  GO:0009862 | 3.0635 | 10.6295 | 14.8405 | 1.5602 | 1.7688 |
| Cre07.g314500^ab^ | GO:0009751 | 6.3416 | 3.6545 | 3.5660 | -0.6699 | -1.5959 |
| Cre07.g328900^b^ | GO:0009697 GO:0009862 | 80.2652 | 78.4462 | 38.4683 | 0.0302 | -1.6318 |
| Cre12.g507450^b^ | GO:0009863 | 14.7642 | 8.9101 | 2.7683 | -0.5923 | -3.0127 |
| Cre17.g696950^ab^ | GO:0009751 | 17.9059 | 13.7909 | 2.2715 | -0.2499 | -2.3697 |
| Cre17.g713025^b^ | GO:2000031 | 0.3424 | 0.3447 | 4.1356 | -0.0331 | 1.9215 |
| Cre01.g034350^b^ | GO:2000031 | 0.0477 | 0.1590 | 1.4170 | -- | 3.6959 |
| Cre02.g081950^ab^ | GO:0009863 | 6.4151 | 4.1918 | 2.4223 | -0.5605 | -2.0397 |

Abbreviations: a: genes related to hormone were enriched to signal transducer activity in molecular function. b: genes related to hormone were enriched to signaling biological process. c: the value of FPKM was the average of different experiments. --: DESeq_log2FC was extremely low. Red mark indicated that genes were up-regulated, green mark indicated that genes were down-regulated under the lead stress. Go annotation was carried out based on all plants’ database.GO:0009737: response to abscisic acid; GO:0009751: response to salicylic acid; GO:0009697: salicylic acid biosynthetic process; GO:0009862: salicylic acid mediated signaling pathway; GO:0009863: salicylic acid mediated signaling pathway; GO:2000031: regulation of salicylic acid mediated signaling pathway.

**Table S4.** Up- and down-regulated genes putatively involved in gibberellin.

| Gene ID | Annotation |  | FPKM^c^ |  | DESeq_log2FC | |
| --- | --- | --- | --- | --- | --- | --- |
|  |  | 0 | 3 | 80 | 0vs3 | 0vs80 |
| Cre01.g009575 | GO:0009686 | 0.6644 | 1.2725 | 12.0093 | 0.9595 | 3.5275 |
| Cre01.g034350^b^ | GO:0009739 | 0.0477 | 0.1590 | 1.4170 | -- | 3.6959 |
| Cre05.g234656^b^ | GO:0009686 | 0.0309 | 0.0353 | 0.4634 | -- | 3.2850 |
| Cre06.g275350 | GO:0009739 | 0.1335 | 0.2645 | 0.9044 | 1.0158 | 2.1599 |
| Cre09.g390000^b^ | GO:0009739 | 0.0882 | 0.0651 | 1.1557 | -- | 3.0566 |
| Cre16.g647602^b^ | GO:0009739 | 0.3382 | 0.5212 | 3.0983 | 0.7655 | 1.7744 |

Abbreviations: a: genes putatively related to hormone were enriched to signal transducer activity in molecular function. b: genes putatively related to hormone were enriched to signaling biological process. c: the value of FPKM was the average of different experiments. --: DESeq_log2FC was extremely low. Red mark indicated that genes were up-regulated, green mark indicated that genes were down-regulated under the lead stress. Go annotation was carried out based on all plants’ database. GO:0009686: putatively gibberellin biosynthetic process; GO:0009739: putatively response to gibberellin.

**Table S5.** Up- and down-regulated genes putatively involved in cytokinin.

| Gene ID | Annotation | FPKM^c^ | | | DESeq_log2FC | |
| --- | --- | --- | --- | --- | --- | --- |
|  |  | 0 | 3 | 80 | 0vs3 | 0vs80 |
| Cre01.g002350^b^ | GO:0009735 | 0.9029 | 0.7353 | 8.9337 | -0.2391 | 2.6757 |
| Cre06.g251250^b^ | GO:0009885 | 1.5047 | 7.3694 | 9.8517 | 2.1864 | 1.7415 |
| Cre06.g284400^b^ | GO:0009736 | 0.1445 | 0.0199 | 2.0565 | -- | 3.1036 |
| Cre07.g314650 | GO:0009691 | 2.5254 | 3.2516 | 18.1283 | 0.4168 | 2.2162 |
| Cre07.g340900 | GO:0009691 | 2.2756 | 2.0966 | 12.7425 | -0.2072 | 1.8482 |
| Cre09.g390000^b^ | GO:0009736 | 0.0882 | 0.0651 | 1.1557 | -- | 3.0566 |
| Cre09.g402450^b^ | GO:0009735 | 8.5450 | 7.4782 | 58.6353 | -0.1217 | 2.1500 |
| Cre16.g647602^b^ | GO:0009736 | 0.3382 | 0.5212 | 3.0983 | 0.7655 | 1.7744 |

Abbreviations: a: genes putatively related to hormone were enriched to signal transducer activity in molecular function. b: genes putatively related to hormone were enriched to signaling biological process. c: the value of FPKM was the average of different experiments. --: DESeq_log2FC was extremely low. Red mark indicated that genes were up-regulated, green mark indicated that genes were down-regulated under the lead stress. Go annotation was carried out based on all plants’ database. GO:0009735: putatively response to cytokinin; GO:0009885: putatively transmembrane histidine kinase; GO:0009736: putatively cytokinin receptor activity cytokinin-activated signaling pathway; GO:0009691: putatively cytokinin biosynthetic process.

**Table S6.** Up- and down-regulated genes putatively involved in auxin.

| Gene ID | Annotation | FPKM^c^ | | | DESeq_log2FC | |
| --- | --- | --- | --- | --- | --- | --- |
|  |  | 0 | 3 | 80 | 0vs3 | 0vs80 |
| Cre03.g199050^b^ | GO:0010540 | 0.3341 | 0.2281 | 2.3465 | -0.4402 | 2.1945 |
| Cre03.g202113 | GO:0009733 | 1.0064 | 1.1712 | 6.3606 | 0.4221 | 2.1882 |
| Cre04.g228300 | GO:0009733 | 6.5289 | 6.1785 | 2.6798 | -0.0166 | -1.8891 |
| Cre06.g275350 | GO:0009733 | 0.1335 | 0.2645 | 0.9044 | 1.0158 | 2.1599 |
| Cre06.g306601^b^ | GO:0009733  GO:0009926 | 3.0635 | 10.6295 | 14.8405 | 1.5602 | 1.7688 |
| Cre07.g325734^b^ | GO:0009734 | 43.3669 | 49.4707 | 15.7315 | 0.2246 | -2.0953 |
| Cre07.g328900^b^ | GO:0009733 | 80.2652 | 78.4462 | 38.4683 | 0.0302 | -1.6318 |
| Cre07.g340900 | GO:0009733 | 2.2756 | 2.0966 | 12.7425 | -0.2072 | 1.8482 |
| Cre09.g390000^b^ | GO:0009733 | 0.0882 | 0.0651 | 1.1557 | -- | 3.0566 |
| Cre09.g394436 | GO:0009926 | 0.4738 | 0.6022 | 10.5408 | 0.3929 | 3.7016 |
| Cre16.g647602^b^ | GO:0009733 | 0.3382 | 0.5212 | 3.0983 | 0.7655 | 1.7744 |
| Cre17.g719450 | GO:0009733 | 11.8935 | 9.9051 | 4.1241 | -0.2136 | -2.1451 |
| Cre01.g002350^b^ | GO:0009734 | 0.9029 | 0.7353 | 8.9337 | -0.2391 | 2.6757 |
| Cre01.g034350^b^ | GO:0009733 | 0.0477 | 0.1590 | 1.4170 | -- | 3.6959 |
| Cre02.g083354 | GO:0009926 GO:0010315 GO:0010328 GO:0010329 | 0.2407 | 0.1613 | 2.9026 | -- | 2.8798 |
| Cre03.g177053 | GO:0009733 | 0.5214 | 0.4762 | 4.0082 | -0.0625 | 2.3203 |
| Cre03.g177150^b^ | GO:0009733 | 0.0592 | 0.0482 | 0.7957 | -- | 2.9443 |

Abbreviations: a: genes putatively related to hormone were enriched to signal transducer activity in molecular function. b: genes putatively related to hormone were enriched to signaling biological process. c: the value of FPKM was the average of different experiments. --: DESeq_log2FC was extremely low. Red mark indicated that genes were up-regulated, green mark indicated that genes were down-regulated under the lead stress. Go annotation was carried out based on all plants’ database.GO:0010540: putatively basipetal auxin transport; GO:0009733: putatively response to auxin; GO:0009926: putatively auxin polar transport; GO:0009734: putatively auxin-activated signaling pathway; GO:0010315: putatively auxin efflux; GO:0010328: putatively auxin influx transmembrane transporter activity; GO:0010329: putatively auxin efflux transmembrane transporter activity.

**Table S7.** Up- and down-regulated genes putatively involved in abscisic acid.

| Gene ID | Annotation | FPKM^c^ | | | DESeq_log2FC | |
| --- | --- | --- | --- | --- | --- | --- |
|  |  | 0 | 3 | 80 | 0vs3 | 0vs80 |
| Cre01.g009575 | GO:0009737 | 0.6644 | 1.2725 | 12.0093 | 0.9595 | 3.5275 |
| Cre01.g026450 | GO:0009737 | 3.4575 | 5.5048 | 17.5092 | 0.7817 | 1.6915 |
| Cre01.g034350^b^ | GO:0009737 | 0.0477 | 0.1590 | 1.4170 | -- | 3.6959 |
| Cre02.g081950^ab^ | GO:0009738 | 6.4151 | 4.1918 | 2.4223 | -0.5605 | -2.0397 |
| Cre02.g087750^b^ | GO:0009737 | 3.4727 | 3.2942 | 1.5082 | -0.0200 | -1.8090 |
| Cre02.g095143^b^ | GO:0071215 | 0.0367 | 0.0256 | 0.3180 | -- | 2.4297 |
| Cre02.g096000 | GO:0009737 | 20.7857 | 14.2702 | 3.4498 | -0.4283 | -2.8936 |
| Cre02.g111500^b^ | GO:0009737 | 0.0957 | 0.0610 | 4.6435 | -- | 4.9975 |
| Cre03.g159254 | GO:0009737 | 0.0095 | 0.0000 | 0.7367 | -- | 5.6522 |
| Cre03.g186950 | GO:0009737 | 0.4348 | 0.2916 | 3.2766 | -- | 2.2507 |
| Cre04.g226550 | GO:0009737 | 2.8801 | 2.5759 | 1.2596 | -0.0902 | -1.7984 |
| Cre05.g236250^b^ | GO:0071215 | 3.7294 | 4.0158 | 0.8439 | -0.1129 | -2.2912 |
| Cre06.g251250^b^ | GO:0071215 | 1.5047 | 7.3694 | 9.8517 | 2.1864 | 1.7415 |
| Cre06.g275350 | GO:0009737 | 0.1335 | 0.2645 | 0.9044 | 1.0158 | 2.1599 |
| Cre06.g287000 | GO:0009737 | 0.1106 | 0.1943 | 1.2971 | -- | 2.9491 |
| Cre06.g306601^b^ | GO:0009738 | 3.0635 | 10.6295 | 14.8405 | 1.5602 | 1.7688 |
| Cre06.g310100^b^ | GO:0009738 | 62.9647 | 52.3425 | 21.3814 | -0.1783 | -2.0477 |
| Cre07.g328900^b^ | GO:0009789 | 80.2652 | 78.4462 | 38.4683 | 0.0302 | -1.6318 |
| Cre08.g384250^b^ | GO:0009737 | 4.7525 | 2.4106 | 1.9905 | -0.9024 | -1.8673 |
| Cre09.g390000^b^ | GO:0009788 | 0.0882 | 0.0651 | 1.1557 | -- | 3.0566 |
| Cre09.g392350 | GO:0009737 | 4.3071 | 7.1340 | 42.8135 | 0.7484 | 2.6434 |
| Cre09.g401022^b^ | GO:0009738 | 0.0000 | 0.0000 | 3.0283 | -- | Inf |
| Cre10.g428200 | GO:0009737 | 6.6969 | 10.1416 | 39.9580 | 0.7847 | 1.9675 |
| Cre10.g452250 | GO:0009737 | 218.6027 | 313.6803 | 0.7823 | 0.5646 | -8.6897 |
| Cre12.g485600 | GO:0009737 | 30.4217 | 27.8853 | 17.7679 | -0.0768 | -1.3951 |
| Cre12.g507450^b^ | GO:0009737 | 14.7642 | 8.9101 | 2.7683 | -0.5923 | -3.0127 |
| Cre12.g540650 | GO:0009737 | 55.9874 | 46.1050 | 21.2269 | -0.2476 | -1.8693 |
| Cre12.g560300 | GO:0009737 | 0.6397 | 0.4509 | 4.6882 | -0.3832 | 2.2834 |
| Cre14.g612500 | GO:0009737 GO:0009788 | 0.3692 | 0.0416 | 4.6161 | -- | 2.9740 |
| Cre16.g647602^b^ | GO:0009788 | 0.3382 | 0.5212 | 3.0983 | 0.7655 | 1.7744 |
| Cre16.g657350^b^ | GO:0009738 | 51.9627 | 57.6302 | 30.1192 | 0.2383 | -1.5289 |
| Cre16.g665364^b^ | GO:0009789 | 82.6277 | 74.7630 | 46.2340 | -0.0589 | -1.4410 |
| Cre16.g684800^b^ | GO:0009737 | 0.2646 | 0.1277 | 2.4443 | -- | 2.3081 |
| Cre17.g696950^ab^ | GO:0009738 | 17.9059 | 13.7909 | 2.2715 | -0.2499 | -2.3697 |
| Cre17.g713025^b^ | GO:0009788 | 0.3424 | 0.3447 | 4.1356 | -0.0331 | 1.9215 |
| Cre17.g717050 | GO:0009737 GO:0080168 | 2.4806 | 1.8501 | 0.7974 | -0.3619 | -2.2678 |

Abbreviations: a: genes putatively related to hormone were enriched to signal transducer activity in molecular function. b: genes putatively related to hormone were enriched to signaling biological process. c: the value of FPKM was the average of different experiments. --: DESeq_log2FC was extremely low. Red mark indicated that genes were up-regulated, green mark indicated that genes were down-regulated under the lead stress. Go annotation was carried out based on all plants’ database.GO:0009737: putatively response to abscisic acid; GO:0009738: putatively abscisic acid-activated signaling pathway; GO:0071215: putatively cellular response to abscisic acid; GO:0009789: putatively stimulus positive regulation of abscisic acid-activated signaling pathway; GO:0009788: putatively negative regulation of abscisic acid-activated signaling pathway; GO:0080168: putatively abscisic acid transport.

**Table S8.** Up- and down-regulated genes putatively involved in ethylene.

| Gene ID | Annotation | FPKM^c^ | | | DESeq_log2FC | |
| --- | --- | --- | --- | --- | --- | --- |
|  |  | 0 | 3 | 80 | 0vs3 | 0vs80 |
| Cre01.g034350^b^ | GO:0009723 | 0.0477 | 0.1590 | 1.4170 | -- | 3.6959 |
| Cre02.g081950^ab^ | GO:0009723 | 6.4151 | 4.1918 | 2.4223 | -0.5605 | -2.0397 |
| Cre02.g111500^b^ | GO:0009723 | 0.0957 | 0.0610 | 4.6435 | -- | 4.9975 |
| Cre03.g203500 | GO:0009723 | 0.0635 | 0.0968 | 1.9130 | -- | 2.6411 |
| Cre05.g234656^b^ | GO:0009723 | 0.0309 | 0.0353 | 0.4634 | -- | 3.2850 |
| Cre06.g251250^b^ | GO:0009723 | 1.5047 | 7.3694 | 9.8517 | 2.1864 | 1.7415 |
| Cre06.g254917 | GO:0009723 | 0.0000 | 0.0009 | 0.8675 | -- | Inf |
| Cre06.g275350 | GO:0009723 | 0.1335 | 0.2645 | 0.9044 | 1.0158 | 2.1599 |
| Cre06.g306601^b^ | GO:0009723 | 3.0635 | 10.6295 | 14.8405 | 1.5602 | 1.7688 |
| Cre06.g310100^b^ | GO:0009723 | 62.9647 | 52.3425 | 21.3814 | -0.1783 | -2.0477 |
| Cre07.g328900^b^ | GO:0009723 | 80.2652 | 78.4462 | 38.4683 | 0.0302 | -1.6318 |
| Cre08.g385350^b^ | GO:0009873 | 133.2668 | 129.0950 | 37.0089 | 0.0047 | -2.4739 |
| Cre09.g391356^b^ | GO:0009723 | 0.0462 | 0.0357 | 0.6169 | -- | 3.0760 |
| Cre09.g401022^b^ | GO:0009723 | 0.0000 | 0.0000 | 3.0283 | -- | Inf |
| Cre10.g422900^b^ | GO:0009723 | 29.1963 | 27.7383 | 16.7281 | -0.0158 | -1.4266 |
| Cre10.g423200^b^ | GO:0009723 | 5.4310 | 3.6392 | 3.5114 | -0.5620 | -1.6691 |
| Cre11.g483000^ab^ | GO:0009723 | 5.3518 | 3.7826 | 0.9063 | -0.3721 | -3.1217 |
| Cre12.g553700 | GO:0009723 | 24.1206 | 26.3812 | 142.3002 | 0.1585 | 1.9324 |
| Cre17.g713025^b^ | GO:0009723 | 0.3424 | 0.3447 | 4.1356 | -0.0331 | 1.9215 |
| Cre17.g739650 | GO:0071369 | 12.0732 | 10.9503 | 4.7195 | -0.0992 | -1.9749 |

Abbreviations: a: genes putatively related to hormone were enriched to signal transducer activity in molecular function. b: genes putatively related to hormone were enriched to signaling biological process. c: the value of FPKM was the average of different experiments. --: DESeq_log2FC was extremely low. Red mark indicated that genes were up-regulated, green mark indicated that genes were down-regulated under the lead stress. Go annotation was carried out based on all plants’ database. GO:0009723: putatively response to ethylene; GO:0009873: putatively ethylene-activated signaling pathway cellular; GO:0071369: putatively response to ethylene stimulus.

**Table S9.** Up- and down-regulated genes putatively related to transcription factors.

| Gene ID | Annotation | FPKM^c^ | | | DESeq_log2FC | |
| --- | --- | --- | --- | --- | --- | --- |
|  |  | 0 | 3 | 80 | 0vs3 | 0vs80 |
| Cre09.g389750 | C2H2 | 0.9922 | 1.5797 | 11.9132 | 0.7676 | 2.9707 |
| Cre01.g009575 | AP2 | 0.6644 | 1.2725 | 12.0093 | 0.9595 | 3.5275 |
| Cre08.g385350 | AP2 | 133.2668 | 129.0950 | 37.0089 | 0.0047 | -2.4739 |
| Cre01.g034350 | MYB | 0.0477 | 0.1590 | 1.4170 | -- | 3.6959 |
| Cre04.g216204 | bHLH | 0.0086 | 0.0423 | 14.7160 | -- | 10.0482 |
| Cre07.g353555 | bHLH | 0.2122 | 0.1306 | 1.9571 | -0.6360 | 2.5703 |
| Cre16.g667150 | bHLH | 0.1014 | 0.1346 | 1.4724 | -- | 4.3518 |
| Cre14.g631750 | bZIP | 8.3190 | 6.4240 | 2.2231 | -0.2902 | -2.5166 |
| Cre17.g746547 | bZIP | 105.8949 | 133.0862 | 63.4504 | 0.3894 | -1.3701 |
| Cre01.g033900 | SBP | 5.7874 | 5.1715 | 1.6935 | -0.1310 | -2.4578 |
| Cre01.g043550 | SBP | 0.9113 | 0.6650 | 0.5544 | -0.3885 | -2.3733 |
| Cre02.g112750 | SBP | 47.7548 | 57.1435 | 26.5457 | 0.3128 | -1.5291 |
| Cre16.g672300 | YABBY | 1.7752 | 1.2712 | 8.8311 | -0.4276 | 1.6759 |
| Cre08.g358532 | GATA | 1.9564 | 8.7512 | 7.0024 | 2.1439 | -0.6275 |
| Cre08.g358534 | GATA | 12.9210 | 0.0040 | 14.4885 | -11.2988 | -0.5067 |
| Cre03.g200431 | HB-other | 12.0484 | 11.1568 | 6.4095 | -0.0358 | -1.6747 |
| Cre03.g198800 | MYB-related | 0.1159 | 0.2535 | 1.8692 | -- | 4.0172 |
| Cre09.g397475 | MYB-related | 0.0268 | 0.0042 | 2.3321 | -- | 5.4819 |
| Cre14.g621172 | MYB-related | 1.4165 | 1.4940 | 0.6173 | 0.1309 | -1.8213 |
| Cre15.g639350 | MYB-related | 0.0197 | 0.0107 | 0.1912 | -- | 2.5904 |

Abbreviations: c: the value of FPKM was the average of different experiments. --: DESeq_log2FC was extremely low. Red mark indicated that genes were up-regulated, green mark indicated that genes were down-regulated under the lead stress.

**Table S10.** Up- and down-regulated genes putatively involved in glutathione metabolism and phytochelatins metabolism.

| Gene ID | Annotation | FPKM^c^ | | | DESeq_log2FC | |
| --- | --- | --- | --- | --- | --- | --- |
|  |  | 0 | 3 | 80 | 0vs3 | 0vs80 |
| Cre12.g559800 | glutathione metabolism | 0.9314 | 0.9629 | 673.5890 | 0.1044 | 8.8723 |
| Cre17.g742450 | glutathione metabolism | 6.5467 | 10.7724 | 59.2051 | 0.0781 | 1.5186 |
| Cre07.g325748 | glutathione metabolism | 8.6668 | 6.9166 | 60.0756 | -0.2790 | 2.1631 |
| Cre08.g358525 | glutathione metabolism | 17.1724 | 12.1332 | 4.5190 | -0.2596 | -2.5345 |
| Cre14.g629960 | phytochelatins metabolism | 0.1126 | 0.1072 | 0.5507 | -- | 1.6494 |

Abbreviations: c: the value of FPKM was the average of different experiments. --: DESeq_log2FC was extremely low. Red mark indicated that genes were up-regulated, green mark indicated that genes were down-regulated under the lead stress.

**Table S11.** Up- and down-regulated genes putatively related to ABC-transporters.

| Gene ID | FPKM^c^ | | | DESeq_log2FC | |
| --- | --- | --- | --- | --- | --- |
|  | 0 | 3 | 80 | 0vs3 | 0vs80 |
| Cre17.g717050 | 2.4806 | 1.8501 | 0.7974 | -0.3619 | -2.2678 |
| Cre16.g674500 | 0.0609 | 0.0778 | 0.4984 | -- | 2.3275 |
| Cre12.g540650 | 55.9874 | 46.1050 | 21.2269 | -0.2476 | -1.8693 |
| Cre12.g530900 | 125.2042 | 108.7057 | 37.2018 | -0.1380 | -2.3784 |
| Cre04.g228650 | 0.4271 | 0.4746 | 3.5211 | 0.1796 | 2.4189 |
| Cre03.g191350 | 0.0651 | 0.0505 | 1.9204 | -- | 2.6246 |
| Cre02.g097800 | 17.8164 | 17.5189 | 3.5947 | 0.0185 | -2.9390 |
| Cre02.g096000 | 20.7857 | 14.2702 | 3.4498 | -0.4283 | -2.8936 |
| Cre02.g083354 | 0.2407 | 0.1613 | 2.9026 | -- | 2.8798 |
| Cre01.g007000 | 10.9746 | 10.2014 | 3.4895 | -0.0646 | -2.2779 |

Abbreviations: c: the value of FPKM was the average of different experiments. --: DESeq_log2FC was extremely low. Red mark indicated that genes were up-regulated, green mark indicated that genes were down-regulated under the lead stress.
