# Supplementary material for: Impact of Pb on Chlamydomonas reinhardtii at Physiological and Transcriptional Levels
Source: Front Microbiol. 2020 Jun 26;11:1443. doi: 10.3389/fmicb.2020.01443 (PMC7333365; doi:10.3389/fmicb.2020.01443)
Supplement: Supplementary file 2 [file Data_Sheet_2.docx]

Supplementary Material 4


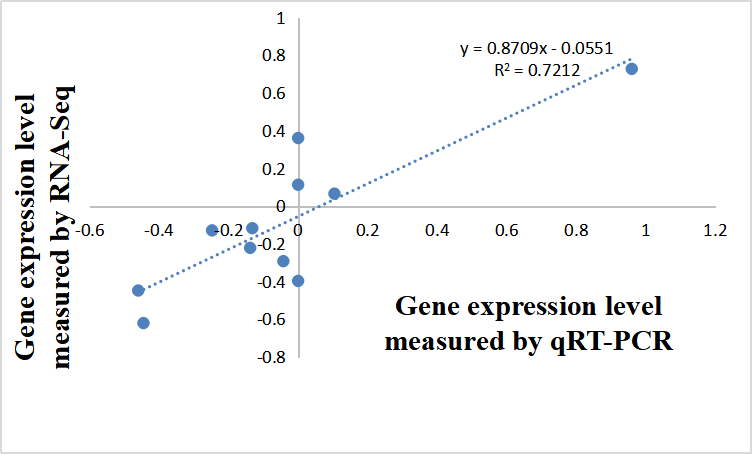


Figure S1. Correlations of expression level analyzed by RNA-Seq platform (y axis) with data resulted from qRT-PCR (x axis) in 0vs3 group.


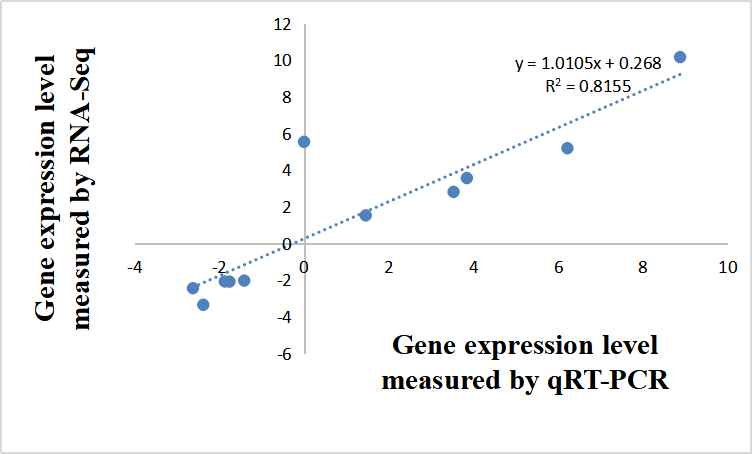


Figure S2. Correlations of expression level analyzed by RNA-Seq platform (y axis) with data resulted from qRT-PCR (x axis) in 0vs80 group.
